# Myosin-actin pattern links matrix stiffness to GFAT2-hyaluronan metabolism

**DOI:** 10.1016/j.mtbio.2025.102305

**Published:** 2025-09-13

**Authors:** Yuwei Zhou, Yifei Zheng, Biao Sheng, Jian Wang, Kefeng Ding, Baohua Ji, Yu Wu

**Affiliations:** a Key Laboratory of Soft Machines and Smart Devices of Zhejiang Province and Department of Engineering Mechanics, Zhejiang University, Hangzhou, 310027, China; b State Key Laboratory of Fluid Power and Mechatronic Systems, Zhejiang University, Hangzhou, 310027, China; c Oujiang Laboratory (Zhejiang Lab for Regenerative Medicine, Vision, and Brain Health) and Wenzhou Institute of University of Chinese Academy of Science, Wenzhou, China; d Department of Colorectal Surgery and Oncology, The Second Affiliated Hospital, Zhejiang University School of Medicine, Hangzhou, Zhejiang, China; e Zhejiang Provincial Clinical Research Center for Cancer, China; f The Fourth Affiliated Hospital of School of Medicine, and International School of Medicine, International Institutes of Medicine, Zhejiang University, Yiwu, 322000, China

**Keywords:** Extracellular matrix, GFPT, Mechanotransduction, Spatial pattern, Breast cancer

## Abstract

While dysregulated extracellular matrix deposition and stiffening are known to drive tumor progression, breast cancer cells can persist, relapse, and metastasize to soft microenvironments. The distinct strategies that tumor cells adapted to soft matrices remain to be further explored. Here, we report that breast tumor cells exploit soft matrices to activate GFAT2-mediated hyaluronan metabolism that can modulate macrophages. This process is driven by the upregulation of GFAT2 expression through enhanced nuclear translocation of NF-κB and XBP1s, coupled with elevated GFAT activity and subsequent hyaluronan production via suppressed AMPKα phosphorylation. Mechanistically, the expression and activity of GFAT2 are jointly modulated by the total cellular levels of myosin and F-actin. More specifically, the ROCK-Rac1 balance, which can regulate both the cortical-to-cytoplasmic ratio of active myosin and the circumferential arrangement of cortical F-actin, mediates the NF-κB-XBP1s-GFAT2 signaling axis. Furthermore, our *in silico* modeling validates that the spatial pattern of myosin directly regulates the orientation of cortical F-actin arrangement. These findings establish a novel mechano-metabolic link between matrix compliance and detrimental mediators—NF-κB/XBP1s, GFAT, and hyaluronan—uncovering a potential microenvironmental reprogramming strategy adopted by tumor cells in soft niches.

## Highlights

• Soft matrices promote GFAT2-HA biosynthesis to reprogram macrophages.
• Soft matrices support GFAT2 expression through NF-κB and XBP1s.
• Total levels of myosin motors and F-actin negatively affect GFAT2.
• Actin ring regulated by myosin pattern links soft matrices to NF-κB/XBP1s-GFAT2.
• Lower levels of p-AMPK on soft matrices support GFAT activity but not stability.

## Graphical abstract

**Figure:**
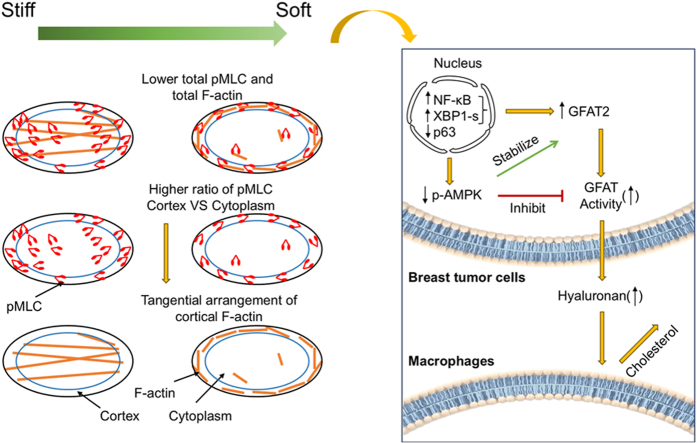

## 1 Introduction

The deposition and cross-linking of extracellular matrix proteins, resulting in increased matrix stiffness or rigidity, commonly serve as one of the pathological diagnostic cues. The growth of solid tumors and the alterations in the surrounding matrix are widely regarded as mutually reinforcing processes [1]. However, there is growing evidence *in vitro* that tumors, particularly breast tumors, take advantage of soft (non-dense) matrices, leading to increased therapy resistance, enhanced stemness [[2], [3], [4], [5], [6], [7]], and immunosuppression [8]. Furthermore, breast cancer in vivo tends to disseminate and relapse in tissue softer than the normal mammary gland or the primary breast tumor [6,7], as well as lurk in softer and more homogeneous tissues following chemotherapy [5]. Although cancer cells typically show less robust function and characteristics on soft matrices, the potential role of matrix compliance in tumor progression merits further investigation, particularly concerning the accumulation of tumor cell potential and the possible support from other cells.

Besides, tumor metabolism can also interfere with the treatment effect and has recently been discovered to be mechanosensitive due to its dependence on the actin cytoskeleton [[9], [10], [11], [12]]. The metabolic changes in tumor cells affect not only themselves but also stromal cells and immune cells, reshaping both the physical and biochemical microenvironment to support malignant progression [[13], [14], [15], [16]]. Hyaluronan (HA) is precisely a metabolite that influences the microenvironment in both physical and biochemical ways, as both a component of the extracellular matrix and a signaling molecule. As a rate-limiting enzyme of the hexosamine biosynthesis pathway (HBP) involved in HA production, glutamine-fructose-6-phosphate aminotransferase (GFAT) plays a significant role in the CD44^high^/CD24^low^ stemness of cancer cells [17], supports mesenchymal breast cancer cells through histone modifications [18], and contributes to epithelial-mesenchymal transition in *in vitro* studies [19,20]. The relationship between nuclear factor kappa B (NF-κB) and XBP1-spliced (XBP1s) with GFAT has gradually become evident [[21], [22], [23]]; however, their specific interactions with GFAT1/2 under various cell types and conditions remain unclear. Again, they are also indicators of malignancy and poor prognosis. NF-κB and XBP1s both impede further treatment and contribute to tumor relapse in the stroma after chemotherapy, which corresponds to the softer matrix, by promoting cell stemness and inhibiting apoptosis [5,24]. Extracellular-bound HA typically acts as a maintainer of physical homeostasis, safeguarding the structure and function of tissues. In contrast, soluble HA exerts a more direct impact on the progression of conditions such as tumors and inflammation. Consistent with the effects of NF-κB, XBP1s, and GFAT, soluble HA secreted by tumor cells promotes tumor progression by polarizing macrophages into an M2-dominant tumor-associated phenotype [15] and supporting chemoresistance [25,26]. Not only *in vitro* experiments but also clinical data suggest that GFAT and HA are both critically implicated in the development of breast tumors, particularly those identified as triple-negative breast cancer (TNBC) [[27], [28], [29]]. These prompt us to consider whether and how GFAT and its product, HA, secreted by tumor cells, are associated with matrix compliance through the involvement of NF-κB and XBP1s.

Here, we outline that matrix stiffness mechano-regulates GFAT2 expression and HA biosynthesis in breast cancer cells through myosin force patterning and cortical actin cytoskeletal reorganization. Specifically, soft matrices promote GFAT2 transcription via NF-κB/XBP1s nuclear translocation while simultaneously regulating GFAT2 protein stability and enzymatic activity through phosphorylated AMP-activated protein kinase (p-AMPK) signaling. The actin ring architecture, governed by elevated phospho-myosin light chain (pMLC) polarization at the cortical region relative to cytoplasmic pools or diminished F-actin polymerization dynamics, contributes to the mechanochemical activation of the NF-κB/XBP1s-GFAT2 axis. Notably, while p-AMPK plays a role in stabilizing the GFAT2 protein, it negatively impacts GFAT activity and consequent secretion of HA. This biophysical regulatory mechanism, active in soft microenvironments such as primary tumors, treatment-remodeled niches, and specific metastatic sites, establishes GFAT2/HA signaling as a biomechanical driver of microenvironmental reprogramming in breast cancer.

## 2 Materials and Methods

### 2.1 Cell culture and preparation of polyacrylamide gels

MDA-MB-231 (RRID:CVCL_0062) and Raw 264.7 (RRID:CVCL_0493) cells were cultured in Dulbecco's Modified Eagle Medium (DMEM) supplemented with 10 % fetal bovine serum (Gibco) and 1 % penicillin-streptomycin (Gibco) at 37 °C with 5 % CO2 and were regularly checked for mycoplasma contamination. Raw 264.7 was passaged without trypsin to prevent differentiation and then checked for cellular shapes before the experiment. MDA-MB-231 were cultured on synthetic polyacrylamide (PA) hydrogels with low (∼1 Kpa) or high (∼20 Kpa) stiffness, as previously described [30]. Formulas with nearly the same crosslinker concentration were chosen for comparable voids and more uniform gels [31]. Briefly, hydrophilic glass coverslips were activated by applying a solution containing acetic acid, 3-(Trimethoxysilyl)propyl methacrylate (Sigma), and ethanol (1:1:14), and washed with ethanol [32]. Gels activated by Sulfo-SANPAH (Sigma-Aldrich) were functionalized with 200 μg/mL Collagen I (Rat Tail, Corning) overnight at 4 °C and rinsed twice with PBS and DMEM before cell seeding. 1 % fluorescent green latex beads (Sigma) were added extra in traction force measurements.

### 2.2 Reagents

Compounds and working concentrations were used as follows: 0.5 μM Latrunculin A, 100 μg/ml Cycloheximide (GlpBio), 50 μM Blebbistatin, 25 μM NSC 23766, 10 μM Y27632, 10 μM Compound C, 0.5 mM AICAR, 20 μM 4μ8C, 10 μM BAY 11–7085 (MCE), 200 μg/ml hyaluronidase (Sigma). Antibodies applied for western blot as follows: polyclonal GFAT1 (RRID:AB_2110155, 1:750), GFAT2 (RRID:AB_2868470, 1:1000), phospho-AMPKα (RRID:AB_331250) and AMPKα (RRID:AB_10622186, 1:1000), β-actin (RRID:AB_2223172, 1:1000). Antibodies used for immunofluorescence as follows: NF-κB p65 (RRID:AB_10859369, 1:500), XBP1s (RRID:AB_2879766, 1:200), p63-α (RRID:AB_2637091, 1:1000), phosphor-myosin light chain 2 (RRID:AB_330248, 1:100). For the calculation of pMLC ratios, actin branches, and ruffle indexes, compounds were applied for 15 min. In other experiments, the compounds were maintained until detection began.

### 2.3 Molecular Biology experiments

Total RNA of cells under paired conditions was extracted simultaneously for contrast using an RNA isolation kit (Omega) following the manufacturer's instructions. Quantitative analysis was performed by the $2^{-\Delta \Delta \text{Ct}\;}$ method with respect to HUWE1 (HECT, UBA and WWE domain containing 1, E3 ubiquitin protein ligase) as the reference gene [27]. The primer sequences utilized in the reactions can be found in Table S2.

For western blot, proteins were separated by SDS-PAGE and transferred onto PVDF membranes (Millipore). The membranes were blocked with 5 % BSA (Solarbio) or 5 % non-fat dry milk (Solarbio) for 1 h at room temperature. Subsequently, the membranes were incubated with the appropriate primary antibody overnight at 4 °C, followed by incubation with an HRP-conjugated secondary antibody (Cell Signaling Technology) for 1 h at room temperature. HRP signals were detected using enhanced chemiluminescence (Biosharp).

### 2.4 GFAT activity assay

To determine the activity of glutamine: fructose-6-phosphate aminotransferase (GFAT), we used a colorimetric method based on the procedure established by Fei Y et al. and R.R. et al. [33,34]. Briefly, cells on PA gels were washed twice with ice-cold PBS and scraped with 120 μl ice-cold GFAT buffer (Tris, 50 mM; EDTA, 5 mM; GSH, 5 mM; glucose-6-phosphate Na2, 5 mM; and KCl, 50 mM). Then the cells were sonicated and centrifuged at 16,000×*g* for 20 min under 4 °C. The resulting supernatants were collected, and the protein concentrations were determined using the Bradford protein assay (Beyotime). The protein concentrations were then adjusted to the same level. Subsequently, 100 μl of the GFAT buffer was mixed with 100 μl of the reactive buffer (F-6-P, 8 mM; glutamine, 6 mM; APAD, 0.3 mM; KCl, 50 mM; KH2PO4, 0.1 mM; GDH, 6 U). The mixture was incubated at 37 °C for 120 min, and the changes in absorbance at 370 nm compared to those without F-6-P were measured.

### 2.5 Hyaluronan measurement

Conditioned medium (CM) from MDA-MB-231 on soft/stiff matrices was collected after 24 h of cultivation. The concentration of hyaluronan was determined using the HA ELISA assay (MEIMIAN) and subsequently normalized to the cell count. 200 μg/ml hyaluronidase at 37 °C for 20 min was applied in CM to eliminate the effects of HA on cholesterol efflux from macrophages.

### 2.6 Lipid raft staining and Flow Cytometry

To stain the lipid rafts, the Vybrant Alexa Fluor 488 Lipid Raft Labeling Kit (ThermoFisher Scientific) was used in accordance with the manufacturer's instructions. In brief, Raw 264.7 cells were gently blown down in DPBS, collected and washed with serum-free DMEM. They were incubated at 4 °C for 10 min with Alexa488-conjugated cholera toxin subunit B (CTB), followed by cross-linking with an anti-CTB antibody for 15 min at 4 °C. Dead cells were excluded using 7-AAD (BD Biosciences) as a gating strategy. We conducted Flow Cytometry using CytoFLEX (RRID:SCR_025067) and analyzed the data with FlowJo cytometric analytical software (RRID:SCR_008520).

### 2.7 Immunofluorescence

Cells fixed with 4 % paraformaldehyde on prepared PA gels were first washed with PBS and then permeabilized with a 0.2 % Triton X-100 solution. After that, they were blocked for 1 h with a 5 % BSA solution in PBS. To visualize F-actin, samples were stained with phalloidin-488 (1:500, Abcam) and DAPI (Invitrogen) for 30 min. To visualize proteins in the nuclei and phosphorylated myosin light chain (pMLC), the samples were incubated with primary antibodies diluted in a 2 % BSA solution overnight at 4 °C. The slides were then washed three times with PBST, incubated with the corresponding secondary antibodies (Cell Signaling, 1:1000) for 1 h at room temperature, and finally stained with a DAPI solution (Invitrogen).

### 2.8 Confocal image analysis

Fluorescence images were obtained using a confocal laser scanning microscope (LSM900, Carl Zeiss). To analyze F-actin characteristics, we captured slices and overlapped stacks in ImageJ (RRID:SCR_003070). We utilized ImageJ plugins, PSF Generator, and DeconvolutionLab2, for image deconvolution. Then, Ridge Detection and Directionality plugins and MATLAB (RRID:SCR_001622) were applied to compile the length of F-actin, F-actin coherence, and the coherence between the predominant direction and the other F-actin, respectively.

The ImageJ magic wand tool was used to select the outlines of cells through summed F-actin images, and the morphological opening was then applied to remove actin branches, allowing for the delineation of the cell edge/boundary. The cell cortex is defined as a shell that begins at the cell boundary and has a thickness of 936 nm. For calculating pMLC ratios between the cellular cortex and cytoplasm, three fluorescence images of pMLC with relatively complete cell contours were summed and then divided by the cortical shell. The mean values of these two regions were then statistically compared. The ruffle index was calculated as the ratio of the area where the F-actin intensity in the cell cortex is greater than one-third of its maximum value to the total area of the cell cortex [35].

To estimate the levels of transcription factors in nuclei, MATLAB software was used to demarcate nuclear contours and extract specific portions of transcription factors from the corresponding binarized nuclei images, slice by slice. The overall levels of transcription factors within the nuclei were quantified using ImageJ, based on the total intensity values of Z-stacks. The schematic diagram, as shown in Fig. S4C, shows the detailed slice-by-slice images of cells on soft/stiff matrices stained with NF-κB and DAPI.

### 2.9 Transfections

Cells were transfected with 20 nM siRNA and performed with RNATransMate (BBI) in an FBS-free culture medium according to the manufacturer's instructions. Sequences of siRNAs are reported in Table S1 (Sangon Biotech). Negative control siRNA was also provided by Sangon Biotech. The effectiveness was verified by immunofluorescence detection (Fig. S4D).

### 2.10 a2-Dimensional TFM

To measure the force applied by cells to the hydrogel surfaces, we captured fluorescence images of cell shapes in the "stressed" state and 500 nm nanobeads within the hydrogel. We then trypsinized the cells from the gels and took a second set of fluorescent images of the beads at specific locations. The paired images were first jitter-corrected with the ImageJ Template Matching plugin, then computationally compared to derive the displacement field within the matrix [36]. To determine the traction force, we applied constrained Fourier transform traction cytometry (FTTC) [37]. Briefly, we used Fourier transform techniques to analyze the spatial frequency components of the deformations, and computed the traction forces based on known material properties and boundary conditions.

### 2.11 Modeling of cells with cortical region and myosin activation with chemo-mechanical feedback

Following the work of Shenoy et al. [38], chemo-mechanical feedback was introduced to the model of cell contractility. The model depicted cells as a fusion of passive mechanical elements and active chemo-mechanical components. The passive component reflected the cytoskeleton's elasticity, and the active part corresponded to the contractile forces produced by phosphorylated myosin motors. These motors would be enhanced by external tension transferred through the connection with the surrounding environments. In the given configuration, the relationships of the stress and the contractile force to the strain were:

[1] $$\begin{array}{c} \sigma_{ij}=\left(\bar{\rho_{0}}+\left(\bar{K}-\frac{2}{3}\bar{\mu }\right)\varepsilon_{kk}\right)\delta_{ij}+2\bar{\mu }\varepsilon_{ij} \end{array}$$

[2] $$\begin{array}{c} \rho_{ij}=\left(\bar{\rho_{0}}+\left(\bar{K_{\rho }}-\frac{2}{3}\bar{u_{\rho }}\right)\varepsilon_{kk}\right)\delta_{ij}+2\bar{u_{\rho }}\varepsilon_{ij}\text{,} \end{array}$$

where the values of parameters were listed in Table S3. The finite element software ABAQUS and the corresponding part UMAT were applied to the model.

### 2.12 Statistical analysis

Statistical analysis was performed and visualized by GraphPad Prism9.5 (RRID:SCR_002798) and Origin 2021. Significances were accepted as p > 0.05. (ns (not significant), ∗(p ≤ 0.05), ∗∗(p ≤ 0.01), ∗∗∗(p ≤ 0.001), and ∗∗∗∗(p ≤ 0.0001)). Statistical comparisons were conducted using three independent experiments.

## 3 Results

### 3.1 Matrix compliance increases GFAT2 and HA secretion

**Fig. 1:**
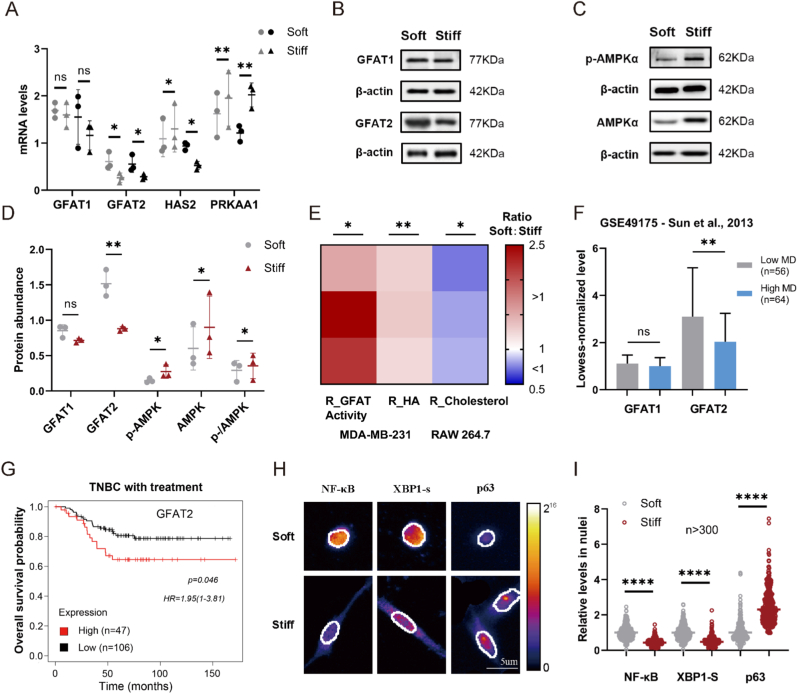
**Soft matrix is sufficient to promote HA secretion through GFAT2 and p-AMPK.** (A) RT-qPCR depicting specified genes in MDA-MB-231 cells cultured on soft or stiff collagen-coated hydrogel matrices for either 10 or 20 h. The gray legend denotes the outcomes of 10 h, and the black one denotes the 20 h results. (B, C) Western blot depicting GFAT1 and GFAT2, phospho-AMPKα, and AMPKα protein levels. β-actin was chosen as a loading control. (D) Quantification of western blot results as shown in (B, C). (E) Relative abundance of GFAT activity and HA from MDA-MB-231, and cholera toxin B (CTB) staining of macrophages (RAW 264.7) with conditioned medium from tumor cells on stiff matrices as compared to soft matrices in a heat map. (F) Lowess-normalized expression levels of GFAT1 and GFAT2 in Sun's datasets for different mammographic densities. (G) Kaplan-Meier curve (Kaplan-Meier Plotter) of patients with TNBC depicting overall survival probability based on GFAT2 gene expression (optimal cutoff by scan). (H) The nuclear edges are marked by white lines, with a scale bar of 5 μm. Fire-type pseudo color indicates the pixel intensity values of NF-κB (3 h), XBP1s (5 h), and p63 (5 h) on soft and stiff matrices. Only the proteins within the outlines of scanned nuclear slices are included in the statistics; the nuclear profiles shown in the image are only their maximum contour. (I) The transcription factors in the nuclei of over 300 cells per condition from 3 biological replicates were quantified by extracting the fluorescence intensity within the nuclei from each scanned image layer and then summing these intensities. Significance: ∗p < 0.05 by ratio paired T-test and Mann-Whitney test for transcription factors in nuclei. Scatter and bar graphs are shown as the mean ± SD of n = 3 biological replicates. (For interpretation of the references to color in this figure legend, the reader is referred to the Web version of this article.)

To examine whether HA production from TNBC cells would respond to mechanical signals, we utilized a recognized *in vitro* framework, where MDA-MB-231 cells were cultured on collagen I-coated polyacrylamide (PA) gels with different stiffness. Collagen absorption on the hydrogels appeared relatively uniform and consistent across different stiffness levels, as assessed by staining and BCA protein assay (Fig. S1). In an attempt to explore the variations in HBP as influenced by the compliance of the matrix, we examined the mRNA, protein levels, and activities of the key rate-limiting enzymes, GFAT1 and GFAT2. Notably, GFAT2 exhibited significant upregulation at both transcriptional and translational levels on the soft matrix (∼1 kPa) compared to those on the stiff one (∼20 kPa), as quantified in Fig. 1A, B, D. In contrast, GFAT1 experienced a slight and slower upregulation under the same mechanical conditions, while HAS2 expression showed biphasic dynamics characterized by transient suppression at early stages followed by compensatory overexpression in later phases (Fig. 1A, B, D).

To functionally characterize GFAT catalytic capacity, we implemented a colorimetric assay measuring the levels of reduced acetylpyridine adenine dinucleotide (APADH) [33]. Consistently, the activity of the enzyme was also enhanced in cells on the soft matrix (Fig. 1E). Since phosphorylation of the energy sensor AMPK is thought to inhibit GFAT enzymatic activity [[39], [40], [41]], we examined AMPK activation status on the two matrices to determine whether matrix stiffness influences factors beyond GFAT expression. MDA-MB-231 cells on the soft matrix showed weaker AMPKα activation, evidenced by less phosphorylated Thr172 and lower total AMPK protein (Fig. 1C and D). This suggests that the soft matrix promotes GFAT activity by inhibiting AMPK. Unlike stromal cells [42,43], MDA-MB-231 cells showed changes in both the phosphorylation level and the expression of the encoding gene, PRKAA1, in response to matrix stiffness (Fig. 1A). Due to the low expression of PRKAA2 in mammary tissue [44], only the influence of PRKAA1 was considered here. While AMPK plays a complex role in tumor development [45], its reduction in cells on the soft matrix and the subsequent effects on the HBP remain noteworthy. Next, we investigated whether the increased GFAT2 levels and GFAT activity not only respond to upstream regulation but also drive HA production.

Consistent with accumulating evidence that UDP-glucose accumulation and HA levels are strongly correlated with GFAT [27], MDA-MB-231 cells on the soft matrix produce more HA, aligning with GFAT performances (Fig. 1E). In addition, elevated HA levels may not only be a consequence of soft matrices but also contribute to malignant tumor progression. Previous studies suggest that conditioned medium (CM) containing higher HA concentrations promotes cholesterol efflux from macrophage membranes, subsequently inducing an M2 phenotype [15,46,47]. We compared the cholesterol-rich membrane microdomains of macrophages cultured in supernatants from MDA-MB-231 cells grown on soft and stiff matrices, finding that CM from the stiff matrix caused less disruption of macrophage lipid rafts (Fig. 1E and S2A), and this difference can be abrogated by hyaluronidase (HAse) treatment (Fig. S2B and C). In addition to the effects of HA on macrophages validated here, HA also has numerous tumor-promoting effects previously reported in other studies [29,48].

Taken together, our findings demonstrate that soft matrices effectively enhance HA synthesis by upregulating GFAT2 expression and augmenting GFAT enzymatic activity, thereby modulating cholesterol efflux of macrophages. This reveals a connection between the physical properties of the extracellular matrix and HA metabolism mediated by the newly discovered environment-sensitive transducer GFAT2. Analysis of two public datasets of human transcriptional profiles from breast cancer patients extends this relationship into an in vivo context. In the TCGA dataset [49], GFAT expression was higher in stage I lesions with the lower modulus (Fig. S2D and E). In a separate dataset [50], tissues surrounding tumors with lower mammographic densities (softer tissue) showed higher GFAT2 expression (Fig. 1F). Furthermore, Kaplan-Meier analysis revealed that GFAT2 expression in TNBC is negatively correlated with survival after chemotherapy, a treatment known to soften the pericellular region (Fig. 1G and S2F). This correlation was not observed in untreated patients (Kaplan-Meier Plotter [51], Fig. S2G), suggesting a potential role of matrix stiffness also in the context of GFAT2 expression to chemotherapy. Next, we investigated the regulatory mechanism by which matrix stiffness influences GFAT2 expression and GFAT activity.

### 3.2 NF-κB and XBP1s link mechanical cues to GFAT2

Considering that NF-κB and XBP1s in solid tumors are primarily regulated by microenvironmental cues rather than genetic alterations [52,53], we examined their nuclear translocation in MDA-MB-231 cells cultured on matrices with different Young's moduli. As shown in Fig. 1H and I, cells on the soft matrix display higher nuclear levels of NF-κB and XBP1s compared to those on stiffer substrates, consistent with GFAT2 levels (Fig. 1A). Based on the close relationship between morphology and NF-κB [54], we analyzed the morphology of MDA-MB-231 cells on soft substrates and found that both cell and nuclear morphology were consistent with higher nuclear NF-κB intensity (Fig. S2H and I). NF-κB has been shown to regulate GFAT2 in some cancers [21,55], while the role of XBP1s in GFAT2 regulation remains less clear [22,56].

**Fig. 2:**
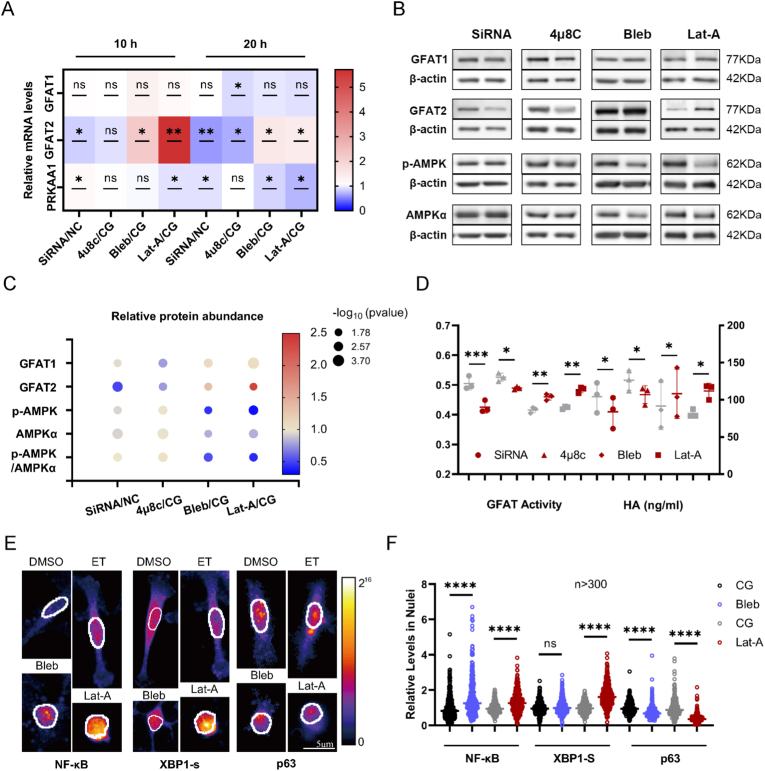
**Transcription factors, myosin contraction, and F-actin elongation are involved in the stiffness-GFAT2-HA signaling axis.** (A) The heatmap illustrates the relative mRNA expression levels of the specified genes under four conditions at 10 h and 20 h: NF-κB silencing, XBP1s inhibition, myosin inhibition, and F-actin inhibition. siRNA for NF-κB silencing and 4μ8C for XBP1s inhibition were applied to cells on the soft matrix; Blebbistatin (Bleb) for myosin inhibition and Latrunculin-A (Lat-A) for F-actin inhibition were employed for cells on the stiff matrix. (B, C) The protein levels of GFAT1, GFAT2, p-AMPK, and AMPK in the aforementioned groups were determined by western blotting. All p-values for the bubble plots are less than 0.05 and are shown by $-\log_{10}\left(pvalue\right)$. (D) GFAT activities and concentrations of HA from cells with the above treatments. (E) The nuclear edges and pixel intensity values of three transcription factors in cells treated with Bleb and Lat-A on stiff matrices are marked by white lines and pseudo color. (F) More than 300 cells (as shown in Fig. E) were quantified relative to the control group (CG), using samples from 3 independent biological replicates. ∗, p < 0.05 according to the ratio paired T-test and Mann-Whitney test for transcription factors in nuclei. Bubble and scatter plots with the mean ± SD are compiled for n = 3 biological replicates. (For interpretation of the references to color in this figure legend, the reader is referred to the Web version of this article.)

To understand the connection between NF-κB and GFAT, we inhibited NF-κB nuclear translocation using Bay 11–7085 and suppressed its expression by transfecting small interfering RNA (siRNA). Surprisingly, siRNA targeting NF-κB was sufficient to inhibit GFAT2, but not GFAT1, gene and protein expression (Fig. 2A–C), but Bay 11–7085 positively regulated GFAT at both levels (Fig. S3A and B). We basically inferred that this drug had the opposite effect due to the promotion of XBP1s [57]. To identify the role of XBP1s in regulating GFAT, we pharmacologically inhibited IRE1α endonuclease activity with 4μ8C to suppress XBP1 splicing. The decreases in both GFAT1 and GFAT2 mRNA and protein levels demonstrated that XBP1s is required for matrix stiffness-affected HBP (Fig. 2A–C). The inhibitory effects of silenced NF-κB and reduced XBP1 splicing also extended to GFAT activity and HA production (Fig. 2D). However, neither PRKAA1, AMPKα, nor phosphorylation of AMPKα was significantly affected by NF-κB and XBP1s modulation (Fig. 2A–C). This indicates that the effects of NF-κB and XBP1s on GFAT activity are independent of p-AMPKα.

To summarize, NF-κB and XBP1s respond to matrix stiffness and influence HA production by regulating GFAT expression, particularly GFAT2. Additionally, this effect is independent of the impact of p-AMPK on GFAT activity.

### 3.3 Traction force and F-actin polymerization are involved in stiffness-dependent GFAT2 modulation

**Fig. 3:**
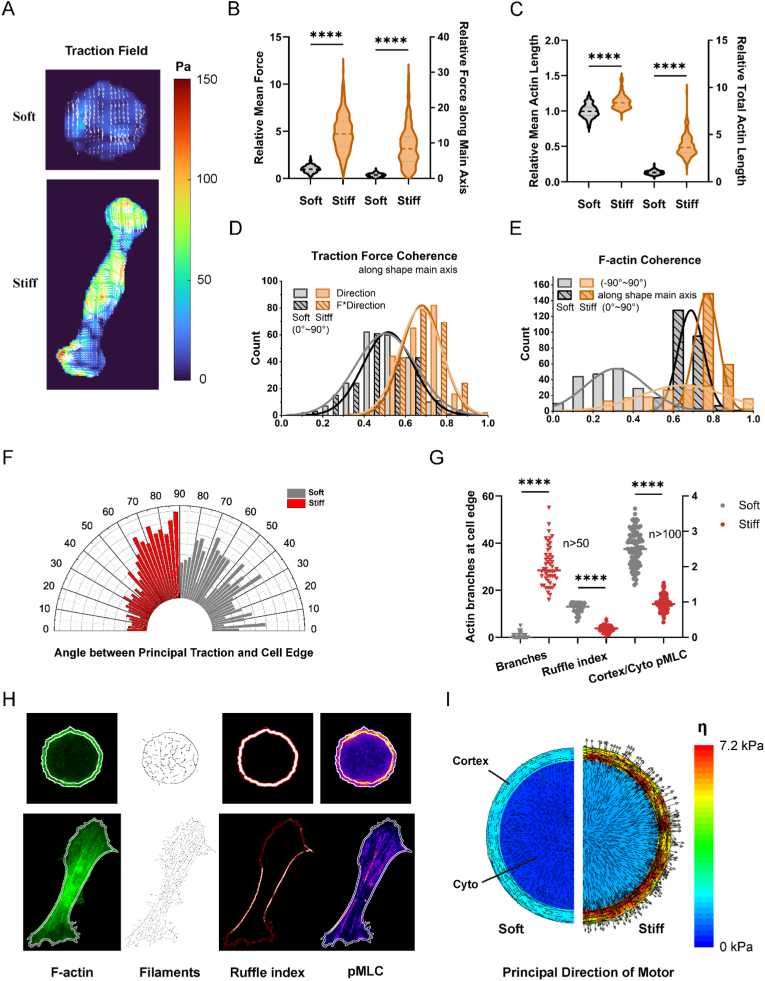
**Characteristics of myosin II and F-actin in MDA-MB-231 on soft and stiff matrices.** (A) Representative images of traction fields of cells on soft and stiff matrices using a traction force microscope. Traction force is presented by color maps, and the in-plane maximum principal stress is displayed by arrows. (B) The relative mean force, ignoring directions and along the main axis of the cell, was statistically analyzed and normalized based on those obtained on soft matrices. (C) Relative mean and total F-actin length of single cells on stiff matrices as compared to those on soft ones, shown as violin plots with median and quartiles. (D) Coherence of traction force direction as a scalar with/without the magnitude of force. (E) Consistency of F-actin orientation distribution on different matrices, with respect to the main axis or not. (F) The angles between principal traction forces and cell boundaries are represented in a histogram ranging from 0° to 90°. The boundary points surrounding the calculation points of the traction force on cell edges were used to calculate the boundary tangent angles. (G, H) Statistical analyses and representative images of F-actin branches, the ruffle index of the cellular cortex, and the ratio of mean phosphorylated myosin light chain (pMLC) levels between the cell cortex and cytoplasm. The cell cortex region does not include fine F-actin branches, as shown by the white lines delineating the cortex region in (H). (I) The simulation of cells on the soft and stiff matrices, where color maps represent the polarization degree of the myosin motor η, and arrows indicate the absolute maximum principal direction and magnitude of myosin contraction. The cell cortex and body were assigned different material properties in the simulation, but these properties remain unchanged across various matrices. ∗, p < 0.05 by unpaired T-test. (For interpretation of the references to color in this figure legend, the reader is referred to the Web version of this article.)

By measuring the displacement of fluorescent particles within the matrix, we obtained the traction force exerted by the cells on the matrix (Fig. 3A). The experimental results showed that cells on the soft matrix exhibited smaller and more uniform principal traction force in both magnitude and directions (Fig. 3B and D). The simulation of single hemispherical cells [59] with cortex could reproduce the traction forces measured on these two matrices (see Materials and Methods for details). Cells on the soft matrix showed lower average motor density $\bar{\rho }=\frac{1}{3}\left(\rho_{1}+\rho_{2}+\rho_{3}\right)$ (Fig. S3F) and lower motor polarization $\eta =\frac{1}{2}\left(\rho_{3}-\rho_{1}\right)$ (Fig. 3I). A flatter and lower position of the nucleus on the stiff matrix, as indicated by logarithmic strain, also aligns with experimental observations (Fig. S3G). Both traction force microscopy and *in silico* modeling showed that cells have higher motor polarization and expend more energy on the stiff matrix, consistent with the observed higher phosphorylation level of AMPK (Fig. 1C). This prompted us to investigate whether myosin II (the active mode and direction of which are estimated by traction force) and subsequent F-actin polymerization affect GFAT.

Myosin inhibitor Blebbistatin (Bleb) increased nuclear levels of NF-κB, but not XBP1s (Fig. 2E and F). Interestingly, GFAT2, but not GFAT1, gene and protein expression continued to follow the trend of NF-κB (Fig. 2A–C), even without the involvement of XBP1s. Given the close connection between traction force and F-actin elongation [60] and parallel alignment [61], we confirmed that the stiff matrix is more conducive to F-actin elongation and parallel orientation, as assessed by average F-actin length in single filaments and individual cells, and coherence distribution (Fig. 3C–E).

To explore whether matrix compliance-mediated regulation of GFAT2 is associated with actin polymerization, we treated MDA-MB-231 cells with Latrunculin A (Lat-A) and monitored GFAT transcriptional and translation activity. As expected, the inhibition of actin polymerization also increased the nuclear translocation of both NF-κB and XBP1s (Fig. 2E and F), as well as the gene and protein levels of GFAT2 (Fig. 2A–C). In contrast to the effects of myosin inhibition, Lat-A also promoted the nuclear translocation of XBP1s, resembling the influence of matrix stiffness on this process.

The disruption of both cellular contractility and actin polymerization markedly impaired AMPK signaling, as evidenced by concomitant reductions in p63-dependent mRNA transcription, protein abundance, and enzymatic activity of AMPK (Fig. 2). These inhibitory effects were associated with enhanced GFAT activity. In conclusion, both Bleb and Lat-A upregulated GFAT through multi-level regulation encompassing enzymatic activation, culminating in enhanced HA secretion (Fig. 2D). These findings establish that attenuated cellular contractility and restrained actin filament polymerization constitute a mechanotransductive determinant of the matrix compliance-dependent GFAT-HA signaling axis.

### 3.4 NF-κB/XBP1s are more closely related to cortical F-actin alignments governed by the spatial distribution of myosin

On the stiff matrix, the maximum principal traction force along the cortex tended to form larger angles with the cell boundary (Fig. 3F), correlating with the number of actin branches at the cell edge (Fig. 3G). We used the ratio of the area where the F-actin intensity in the cell cortex is greater than one-third of its maximum value to the total area of the cell cortex, referred to here as a modified ruffle index [35], to describe the continuity of cortical F-actin (Fig. 3H). Based on the results of actin branches and ruffle indices (Fig. 3G), cells on the soft matrix exhibited more complete and circumferentially arranged F-actin along the cell boundary (actin ring or belt). Concurrently, matrix stiffness modulated the spatial distribution of phosphorylated myosin light chain (pMLC), with soft matrices exhibiting higher cortical-to-cytoplasmic pMLC ratios compared to stiff ones (Fig. 3G and H). The computational model successfully replicated the two synchronized phenomena observed in soft versus stiff matrices. Specifically, cells on the soft matrix exhibited more circumferential alignment of myosin motor dipoles mirroring that of F-actin (Fig. 3I, arrow), and a higher pMLC ratio between the cortex and cytoplasm (Fig. 3I, color), which aligns with experimental results.

**Fig. 4:**
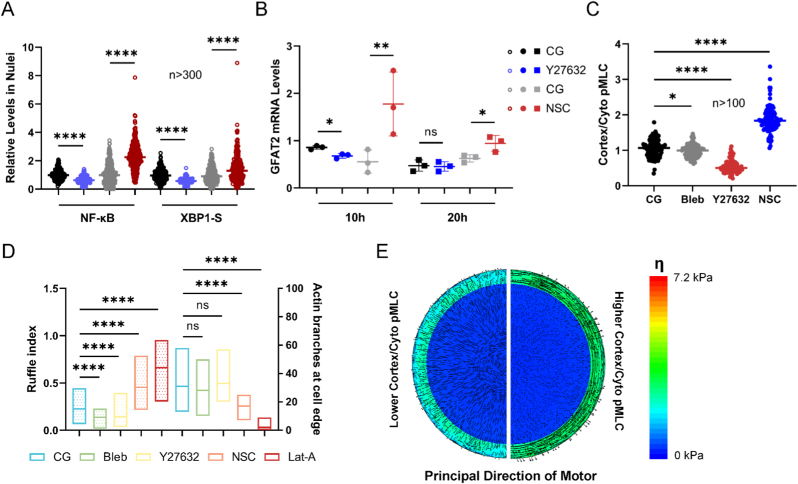
**The direction and distribution of pMLC and F-actin are involved in NF-κB and XBP1s regulating GFAT2.** (A, B) Quantification of transcription factors NF-κB (3 h) and XBP1s (5 h) in scanned nuclei slices of cells and mRNA levels of GFAT2 relative to housekeeping gene treated with either vehicle, Y27632, or NSC23766 (NSC). (C) Mean pMLC ratios between the cortex and cytoplasm were obtained from cells from the control group, the myosin II inhibition, ROCK inhibition, and Rac1 inhibition groups. (D) Ruffle indexes and actin branches at the cell edge for Bleb, Y27632, NSC, and Lat-A treatments. The ruffle index and actin branches are related to the uniformity and arrangement of F-actin distribution along the cell boundary. (E) The simulation of cells with either lower (Bleb or Y27632) or higher (NSC) pMLC ratios between the cortex and cytoplasm, relative to controls cultured on a stiff matrix. The principal directions of motors indicate F-actin alignments. Significance: ∗p < 0.05 by Mann-Whitney test for transcription factors in nuclei and unpaired T-test.

Based on the effects of Bleb, there was a certain discrepancy between the overall inhibition of myosin in cells and the effects of matrix stiffness (Fig. 1, Fig. 2F, XBP1s). Y27632, a ROCK inhibitor that also suppresses total myosin force and subsequent F-actin polymerization, produced opposite effects. It inhibited the nuclear translocation of both NF-κB and XBP1s, as well as the expression of GFAT2 (Fig. 4A and B). When we inhibited another type of Rho GTPases, Rac1, using NSC23766 (NSC), the nuclear translocation of NF-κB and XBP1s, and the expression level of GFAT2 were all opposite to those seen with Y27632 treatment (Fig. 4A and B). Nuclear translocation of NF-κB was still correlated with the shapes of the cells and nuclei (Fig. S4A). While Bleb, Y27632, and NSC all suppressed overall myosin activity, F-actin polymerization, and F-actin coherence (Fig. S4B), their effects on transcription factors diverged significantly. This spectrum of effects, ranging from inhibitory to activating, correlates more strongly with the experimentally observed drug-dependent spatial distributions of active myosin and cortical F-actin alignments (Fig. 4).

The model revealed that solely regulating cortical-to-cytoplasmic pMLC ratios determines the directional bias of force polarization (Fig. 4E), thereby establishing a link between these two phenomena, which can be co-regulated by matrix mechanical properties or pharmacological interventions. Specifically, elevated pMLC ratios promote circumferential alignment of F-actin along the cell periphery, as indicated by the ruffle indexes and the number of F-actin branches (Fig. 4C and D). Overall, NSC enhances the circumferential alignment of cortical forces by increasing the pMLC ratio, thereby facilitating a more circumferential organization of cortical F-actin; Lat-A can also induce the circumferential distribution of cortical F-actin. Given the positive effects of NSC and Lat-A treatments on both the NF-κB/XBP1s-GFAT2 pathway and the actin ring (Fig. 2A and F, 4A-D), we proposed that circumferential alignment of cortical F-actin, rather than overall F-actin coherence or total cellular contractile force, explains the positive regulation of the NF-κB/XBP1s-GFAT2 pathway.

### 3.5 p-AMPK supports GFAT2 stability but not HA secretion

**Fig. 5:**
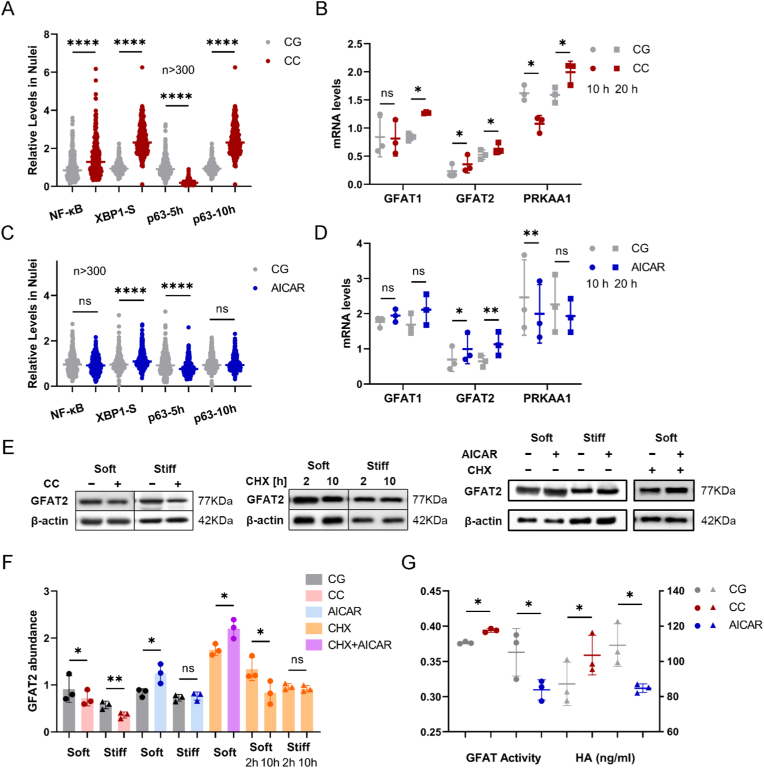
**p-AMPKsupportsGFAT2 stability but not HA secretion.** (A, C) Nuclear levels of NF-κB, XBP1s, and p63 were tested at two different time points in cells treated with Compound C (CC) or AICAR, cells with vehicle treatment serving as the control group (CG). (B, D) RT-qPCR detecting the expression of GFAT1, GFAT2, and prkaa1 in cells with CC or AICAR at 10 and 20 h. (E) Representative results of GFAT2 from cells treated with CC; GFAT2 stability as determined by western blot of cells exposed to CHX for 2 and 10 h; cells treated with AICAR or with AICAR and CHX. β-actin was used as the loading control. (F) Western blot analysis of GFAT2 abundance as depicted in (E). (G) Quantification of GFAT activities and HA concentrations from the same number of cells under CC or AICAR treatments. Bar and scatter graphs show the mean ± SD of three independent experiments.

Phosphorylation of AMPK and its upstream regulators has been reported to inhibit the activity of GFAT1/2 and HAS2 [39,41,62], thereby reducing the production of HA. In cases of a soft matrix, the upregulation of the GFAT2 gene and protein expression, driven by a reduction in F-actin and traction force, along with increased GFAT activity resulting from decreased AMPK levels and phosphorylation due to reduced energy consumption, appears to effectively enhance the overall process from gene expression to HA production. But what is doubtful is that under Bleb and Lat-A treatment, GFAT2, which shows significant differences at the genetic level, does not exhibit further differences at the protein level. To determine whether AMPK plays other roles in the GFAT2-HA process, we inhibited AMPK phosphorylation on the stiff matrix using Compound C (CC) (Fig. S3C). Notably, while this drug encouraged the nuclear entry of NF-κB and XBP1s transcription factors (Fig. 5A) and slightly increased GFAT2 at the transcriptional level on the stiff matrix possessing higher levels of p-AMPK (Fig. 5B), it significantly reduced the protein levels of GFAT2 and showed a similar effect on the soft substrate (Fig. 5E and F).

This greatly exceeded our expectations, so we conducted a reverse attempt to activate AMPK with AICAR in cells on the soft matrix (Fig. S3D). Interestingly, this drug still supported the nuclear translocation of XBP1s, but not NF-κB (Fig. 5C), with an increase in the transcription level of GFAT2, rather than GFAT1 (Fig. 5D). While CC (inhibitor) and AICAR (activator) did not exhibit opposing effects on GFAT2 transcriptional regulation, AICAR treatment ultimately increased GFAT2 protein levels on the soft matrix, and this effect persisted even when cycloheximide (CHX) was used to pharmacologically block de novo protein synthesis, suggesting post-translational regulation (Fig. 5E and F). However, AICAR has no significant effect on GFAT2 in cells on stiff matrices (Fig. 5E and F). The reason the pharmacological activator was less effective at enhancing GFAT2 on the stiff one could be that the p-AMPK presented on this matrix was already enough to keep GFAT2 stable. To explore this further, we monitored the degradation of GFAT2 on different matrices under protein synthesis inhibition by CHX. It was found that GFAT2 levels on the soft matrix dropped significantly, whereas the levels on the stiff one were more stable (Fig. 5E and F). This observation indicated the role of p-AMPKα on GFAT2 stability.

The opposite effects of AMPK on the matrix-GFAT2-HA signaling axis, namely stabilizing GFAT2 protein and inhibiting GFAT and HAS2 activities, sparked our interest in its overall influence on the downstream products of GFAT2. Of note, CC positively influenced the downstream GFAT activities and HA secretion, while AICAR had the opposite effect (Fig. 5G). This highlighted the beneficial interaction of p-AMPK with other factors when comparing GFAT2-HA on soft to stiff matrices. It also provided a glimpse into the complexity of AMPK's role and its effective yet controversial effects in therapy [45,63,64]. Another interesting aspect is how drugs targeting AMPK phosphorylation regulate their own expression. Inhibitors initially promoted AMPK expression through p63, but they suppressed it over time (Fig. 5A and B). Conversely, activator AICAR had a short-term suppressive effect on its expression through p63 (Fig. 5C and D). It is worth noting that the physical properties of the microenvironment can have a more comprehensive impact on the entire process, and a drug may produce opposite effects at different steps in a given process. Each aspect has its benefits, and the interactions between them deserve more investigation. Taken together, while the phosphorylation of AMPK supports the stability of GFAT2, it also acts as an inhibitor in the overall process of matrix compliance-GFAT2-HA.

## 4 Discussion

The survival of early-stage breast cancer often involves interactions with soft microenvironments, including early-stage breast tumor tissue, preferred metastatic sites, and post-chemotherapy residual lesions. While the malignant phenotype characterized by enhanced proliferative and migratory capacities typically manifests on stiff matrices [65,66], growing evidence has begun to delineate how cells adapt to and exploit soft microenvironments [[2], [3], [4], [5], [6], [7]]. For instance, soft matrices enhance cancer cell survival by inducing autophagy to evade anti-estrogen therapies [6] and confer chemotherapy resistance through NF-κB activation and elevated oxidative stress tolerance [5,7]. Despite these advances, the pathophysiological mechanisms underlying soft matrix-driven tumor progression remain incompletely characterized. Here, we demonstrate that breast cancer cells cultured on soft matrices upregulate HA metabolism, which can reprogram macrophages through cholesterol efflux (Fig. 1E) or promote tumor progression via alternative mechanisms [29,48].

NF-κB/XBP1s, GFAT, and HA have been identified as potential malignant indicators associated with the survival and progression of breast cancer [5,24,[27], [28], [29]]. In this work, our data reveal a role of matrix compliance in HA metabolic reprogramming through an increase in GFAT2 expression and GFAT activity. This reveals a mechanochemical link between extracellular matrix properties and HA metabolism mediated by the novel mechanosensitive transducer GFAT2, with neither GFAT1 nor HAS2 exhibiting comparable regulatory effects. In addition to GFAT1's less pronounced response, its lower expression in TNBC cells compared to GFAT2 further underscored its limited biological relevance in this context [67] (Fig. S5A and B). Regarding HAS2 transcriptional regulation, the effects of matrix mechanical properties on HAS2 expression exhibit temporal inconsistency across different time points. We hypothesize that this discrepancy stems from the downstream localization of HAS2 within the HA synthesis signaling pathway: as a downstream effector molecule, the association between mechanical cues and HAS2 expression dynamics is inherently intricate, likely involving multi-layered feedback regulatory loops that modulate its transcriptional activity. Furthermore, in vivo observations have revealed that HAS2 upregulation events are not only infrequent but also show no significant correlation with endogenously measured HA levels [27,48].

On the soft matrix, increased nuclear translocation of NF-κB and XBP1s, along with reduced levels of p-AMPK, contribute to both GFAT2 expression and GFAT activity, respectively. Both NF-κB (through siRNA-mediated knockdown) and XBP1s (via pharmacological inhibition) have been shown to be linked to GFAT2 expression (Fig. 2). Although the NF-κB inhibitor Bay 11–7085 failed to exert the anticipated effect in the present experimental setting (Fig. S3A and B), it is worth noting that this inhibitor has been previously validated to be effective in non-small cell lung cancer [21]. We hypothesize that this inconsistent efficacy may stem from a potential counteractive effect of Bay 11–7085: specifically, this drug has been reported to promote the activation of XBP1s [57], which is highly expressed in breast cancer [58] (Fig. S3E). However, this proposed mechanism remains to be experimentally verified. In contrast to glycolysis [10] but similar to lipid metabolism [12,68], both decreased F-actin polymerization and reduced traction force facilitate GFAT2 expression and HA production by elevating nuclear levels of NF-κB. It is worth noting that the regulation of both NF-κB and XBP1s, with the latter in particular, is more closely related to the balance between ROCK and Rac1, which contributes to both the cortical-to-cytoplasmic pMLC ratio and subsequent cortical F-actin circumferential alignment, rather than simply resulting from the inhibition of total cellular contraction. This is evidenced by the negative, partially positive, and effective regulation of NF-κB/XBP1s-GFAT2 via inhibition of ROCK, myosin II, and Rac1, respectively (Fig. 4). It should be noted that neither the cortical-to-cytoplasmic pMLC ratio nor the subsequent circumferential alignment of cortical F-actin was directly regulated; instead, their regulation was achieved indirectly by modulating ROCK and Rac1.

The link between biophysical stimuli and metabolic pathways has recently begun to attract attention, focusing on key proteins closely related to energy production and consumption [10,42,43]. However, in addition to the metabolic changes that occur in cancer cells, which are crucial for tumor progression, their metabolic byproducts also influence tumor development by altering the surrounding microenvironment. Although inadequate levels of p-AMPK on the soft matrix are not favorable for the stability of the GFAT2 protein, they still enhance GFAT activity and the subsequent production of HA. As a "double-edged sword" in tumors and corresponding therapies [45,63,64], AMPK, which is related to energy metabolism, also exhibits opposing roles in HA metabolism at different stages of the GFAT2-HA process. Here, the soft matrix reduces p-AMPK by decreasing F-actin polymerization and reducing traction forces, thereby facilitating HA production at the level of GFAT activity.

The malignant progression of tumors depends not only on their intrinsic proliferative and migratory capacities but also on active remodeling of cellular and extracellular matrix components within the tumor microenvironment. In this context, upregulation of GFAT2 expression and enzymatic activity on the soft matrix enhances HA secretion and subsequently disrupts membrane cholesterol homeostasis in microenvironmental macrophages. Since the impact of matrix stiffness on the soluble secretions of tumor cells is not limited to HA, and existing literature has already conducted comprehensive studies on the effects of HA on macrophages [15,25], we did not further characterize macrophage phenotypes or other related aspects here.

Specifically, soft matrices facilitate the nuclear translocation of NF-κB and XBP1s while suppressing p-AMPK levels, thereby synchronously enhancing GFAT2 expression, enzyme activity, and HA secretion. This regulatory mechanism is governed by the total levels of active myosin and the corresponding F-actin. Notably, the active myosin pattern and its subsequent actin ring (a circumferentially aligned F-actin structure), which are determined by the ROCK-Rac1 balance, exert a specific influence on the NF-κB/XBP1s-GFAT2 signaling axis. These findings establish a novel link between extracellular matrix stiffness and tumor-stromal cell crosstalk, underscoring the critical role of biophysical stimuli in tumor metabolism via the myosin-actin pattern. While previous investigations have not specifically addressed the mechanical regulation of HA metabolism, NF-κB/XBP1s, GFAT, and HA show correlations with tumor cell preservation of viability in soft matrix-characterized early-stage tumors, preferred metastatic sites, and post-therapeutic residual tissues. This mechanobiological mechanism, mediated through microenvironmental reprogramming of HA biosynthesis, may constitute a novel survival strategy in breast cancer progression. Future investigations will focus on elucidating how the subcellular distribution of pMLC and cortical F-actin networks, along with their downstream mechanotransduction effectors, contribute to tumor adaptive strategies under mechanical stress conditions.

## CRediT authorship contribution statement

**Yuwei Zhou:** Writing – original draft, Visualization, Software, Investigation, Conceptualization. **Yifei Zheng:** Methodology. **Biao Sheng:** Investigation. **Jian Wang:** Resources. **Kefeng Ding:** Resources. **Baohua Ji:** Supervision, Resources. **Yu Wu:** Writing – review & editing, Supervision, Resources, Conceptualization.

## Funding sources

This work was supported by the 10.13039/501100001809National Natural Science Foundation of China (No. 12472035), the 10.13039/501100004731Zhejiang Provincial Natural Science Foundation of China (No. LR20A020001), and the National Natural Science Foundation of China (Nos. 11932017 and 11402227).

## Declaration of competing interest

The authors declare that they have no known competing financial interests or personal relationships that could have appeared to influence the work reported in this paper.

## Data Availability

Data will be made available on request.
