# Supplementary material for: Myosin-actin pattern links matrix stiffness to GFAT2-hyaluronan metabolism
Source: Mater Today Bio. 2025 Sep 13;35:102305. doi: 10.1016/j.mtbio.2025.102305 (PMC12475523; doi:10.1016/j.mtbio.2025.102305)
Supplement: Multimedia component 1 [file mmc1.docx]

**Supplementary Information**

Myosin-actin pattern links matrix stiffness to GFAT2-hyaluronan metabolism

Yuwei Zhou^a^, Yifei Zheng^a,c,#^, Biao Sheng^f,#^, Jian Wang^d,e^, Kefeng Ding^d,e^, Baohua Ji^a^, Yu Wu^a,b,^*

^a^Key Laboratory of Soft Machines and Smart Devices of Zhejiang Province and Department of Engineering Mechanics, Zhejiang University, Hangzhou, 310027, China

^b^State Key Laboratory of Fluid Power and Mechatronic Systems, Zhejiang University, Hangzhou 310027, China

^c^Oujiang Laboratory (Zhejiang Lab for Regenerative Medicine, Vision, and Brain Health) and Wenzhou Institute of University of Chinese Academy of Science, Wenzhou, China

^d^Department of Colorectal Surgery and Oncology, The Second Affiliated Hospital, Zhejiang University School of Medicine, Hangzhou, Zhejiang, China

^e^Zhejiang Provincial Clinical Research Center for Cancer, China

^f^The Fourth Affiliated Hospital of School of Medicine, and International School of Medicine, International Institutes of Medicine, Zhejiang University, Yiwu, 322000, China

#These authors contributed equally to this work

Corresponding author: Yu Wu;

Email: [ywu@zju.edu.cn](mailto:ywu@zju.edu.cn);


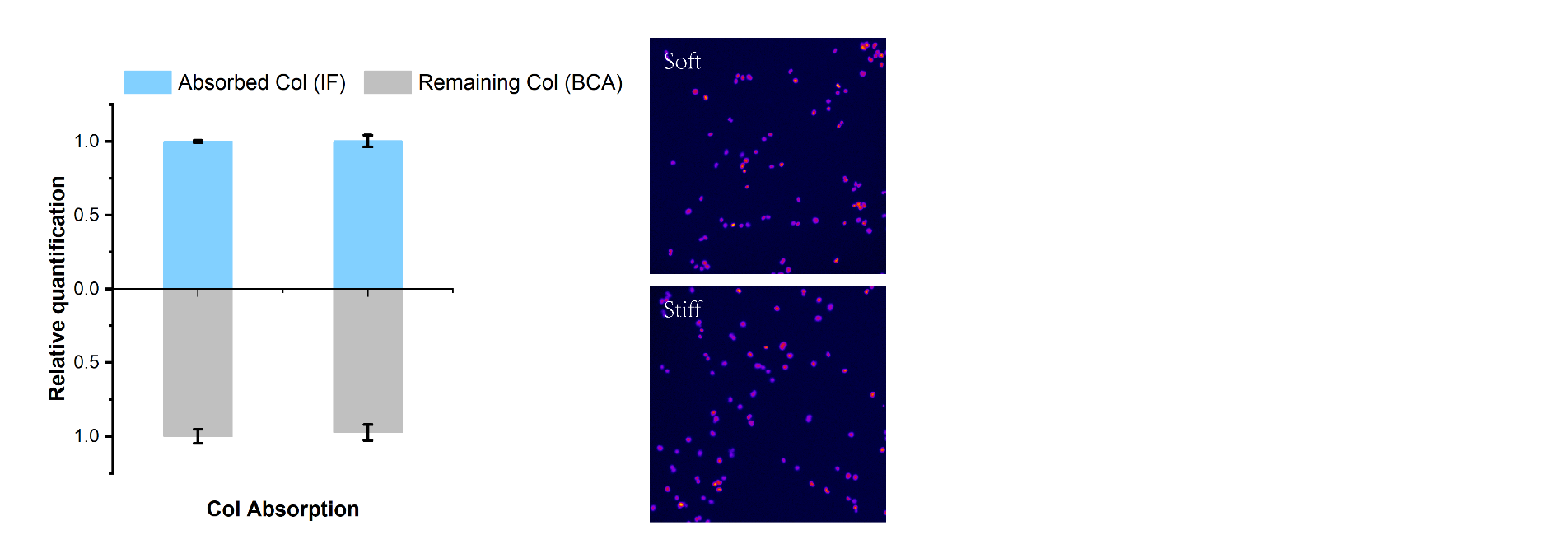
**Fig. S1**

Quantification of mean fluorescence intensities from gel surfaces tagged with anti-collagen antibody, and the amount of collagen in the remaining solution measured by the BCA Protein Assay Kit after overnight incubation.


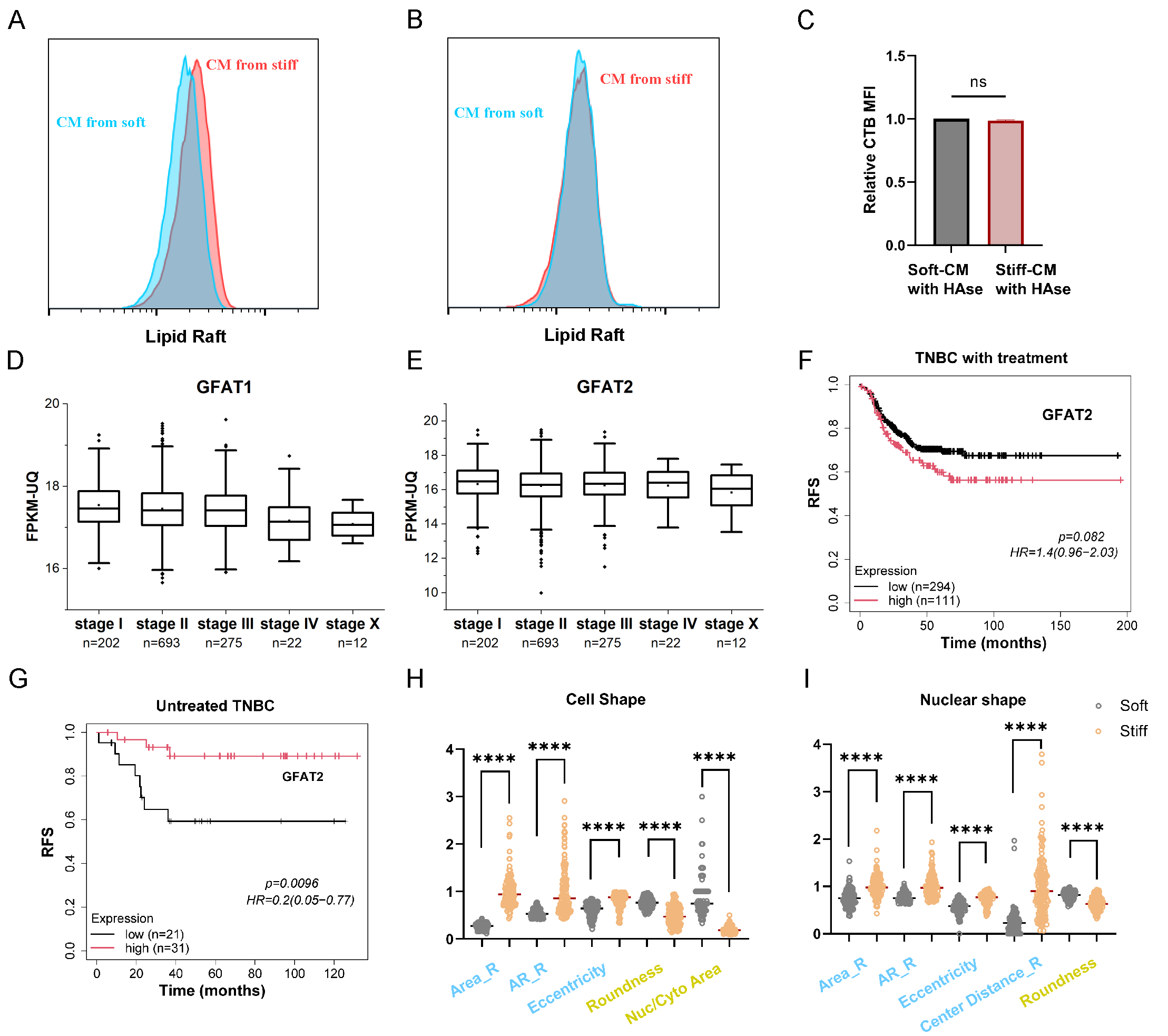
**Fig. S2**

(A) Flow cytometry results of cholera toxin B (CTB)-labeled lipid raft of macrophages treated with condition media (CM) from MDA-MB-231 cells on soft/stiff matrices.

(B, C) Illustration and analysis of CTB results of macrophages with CM supplemented with hyaluronidase (HAse).

(D, E) RNA sequencing data of GFAT1 and GFAT2 from breast cancer samples at different stages (TCGA dataset)[1].

(F, G) Kaplan-Meier curve (Kaplan-Meier Plotter[2]) of TNBC patients with or without therapy depicting recurrence free survival in relation to GFAT2 gene expression (best cutoff by scan).

(H, I) Various cellular and nuclear morphological features associated with the levels of NF-κB in nuclei of MDA-MB-231 cells. Blue text indicates a negative correlation, while yellow text indicates a positive correlation with nuclear NF-κB levels.


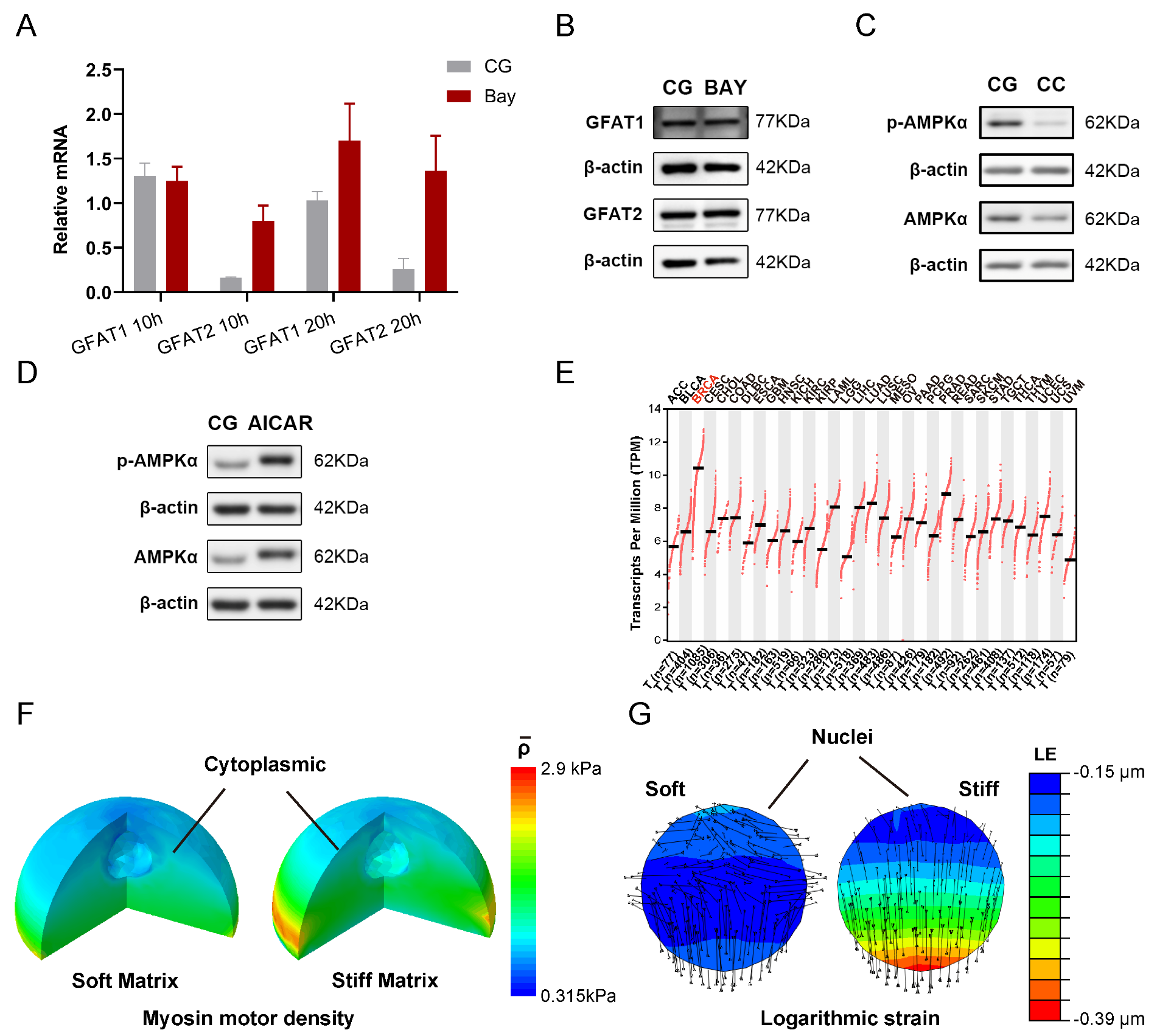
**Fig. S3**

(A, B) Effect of the Bay 11-7085 on GFAT1 and GFAT2 mRNA and protein levels in TNBC cells cultured on the soft matrix. *: P < 0.05, significance by ratio paired t-test. Bar graphs are shown as the mean with S.D of n = 3 biological replicates.

(C, D) Verification of the effects of AMPK inhibitors (Compound C) and agonists (AICAR) on p-AMPK and AMPK, β-actin setting as loading control.

(E) Pan-cancer transcription levels of XBP1 gene in patients with cancer obtained from TCGA projects using a standard processing pipeline[3].

(F) Simulated average myosin motor density in cytoplasm of cells on the soft or stiff matrix.

(G) Simulation of deformed nuclei of cells on the soft or stiff matrix. Color maps represent logarithmic strain and arrows indicate its max principal directions.


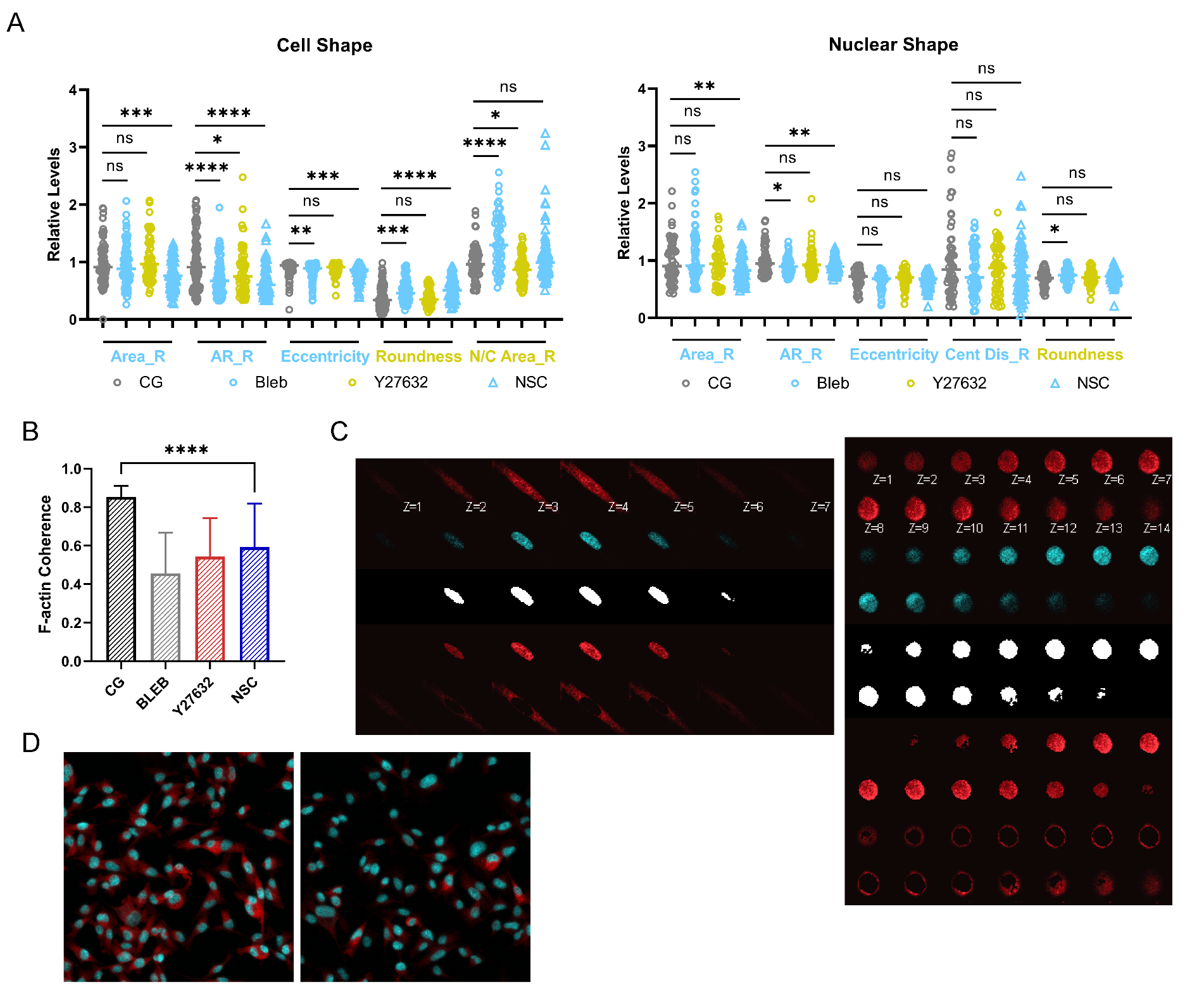
**Fig. S4**

(A) Various cellular and nuclear morphological features associated with the levels of NF-κB in nuclei. Blue text indicates a negative correlation, while yellow text indicates a positive correlation with nuclear NF-κB levels. The blue sample points represent treatments that are positively related to NF-κB nuclear translocation, while the yellow sample points represent the opposite effect.

(B) F-actin coherence of cells with Blebbistatin, Y27632, and NSC 23766 treatments.

(C) Representative pictures of nuclear proteins were quantified. Segmented nuclear and transcription factor images at different Z-sections were captured by a confocal microscope. For the analysis process, segmented nuclear images of cells on soft and stiff matrices were shown and binarized. Levels of transcription factors in the nuclear volume were quantified by extracting the parts that overlap with nuclei. We also showed the cytoplasmic parts of transcription factors.

(D) Diagram showing the silencing effect of siRNA on NF-κB.

**Fig. S**
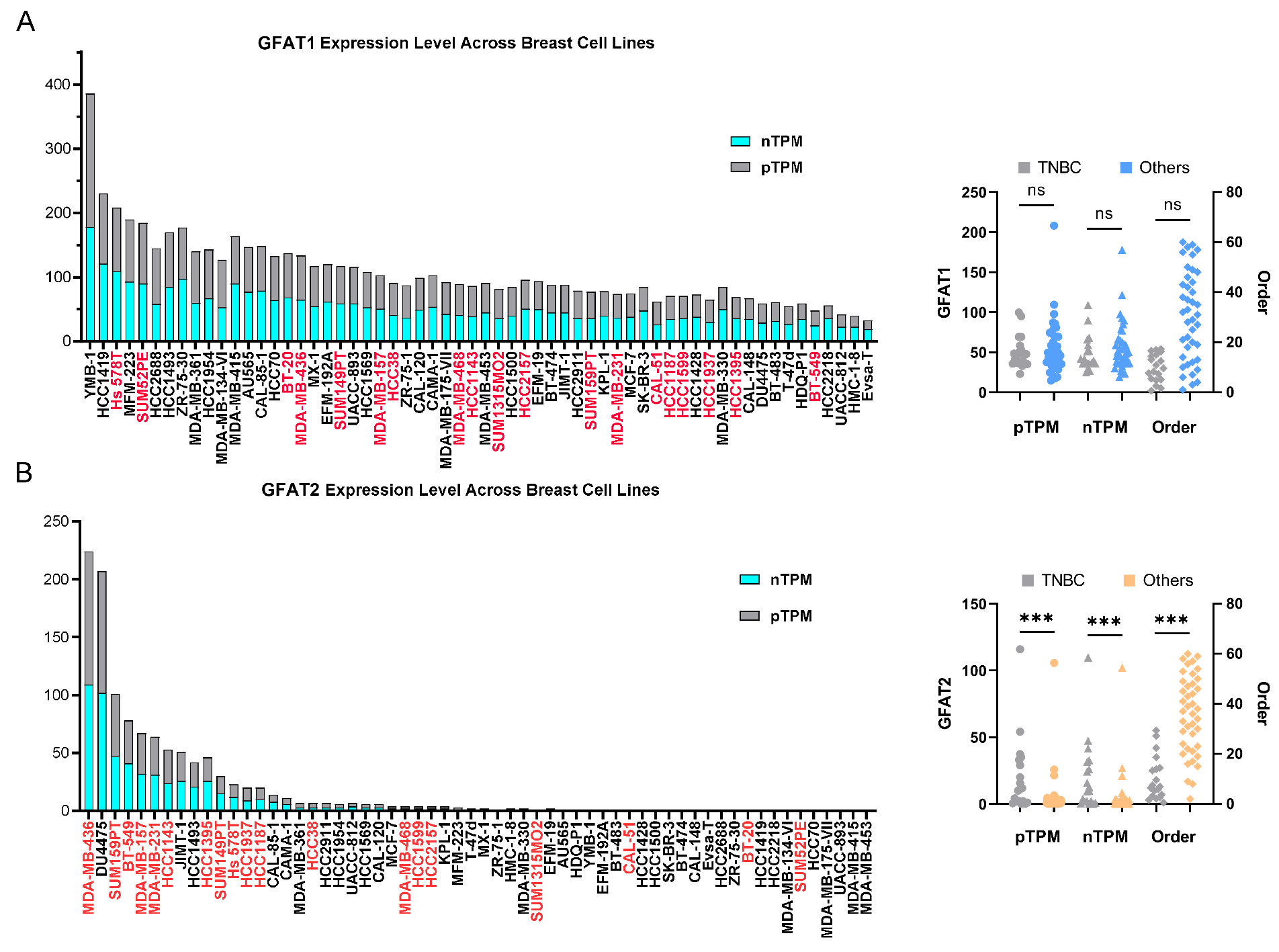
**5**

(A, B) The protein-transcripts per million ("pTPM") and normalized expression ("nTPM") of GFAT1 and GFAT2 in the different breast cancer cell lines used in The Human Protein Atlas[4]. The expression and ranking difference between the TNBC cell line and other cell lines.

**Table S1: siRNA sequences (targeting human genes)**

| Name | Target sequence |  |
| --- | --- | --- |
| hP65_A | CGGAUUGAGGAGAAACGUAAA | UUUACGUUUCUCCUCAAUCCG |
| hP65_B | CCUGAGGCUAUAACUCGCCUA | UAGGCGAGUUAUAGCCUCAGG |
| hP65_C | GCAGGCUAUCAGUCAGCGCAU | AUGCGCUGACUGAUAGCCUGC |

**Table S2: RT-qPCR primer sequences (human)**

| GENE | FORWARD PRIMER | REVERSE PRIMER |
| --- | --- | --- |
| GFAT1 | ACAATCGGGAAAGTCAAGATACC | CACCAATCAACAGAGGGCTAC |
| GFAT2 | ATTCAGCAGTTGGAAGGTG | CTCTTCATCCGTGTCTTACAG |
| HAS2 | GTTGCATGAGTTTGTGGAAGA | TCTCGGAAGTAGGACTTGCTC |
| PRKAA1 | TCAGGAAGATTGTATGCAGGCCCA | TTCATGGGATCCACCTGCAGCATA |
| HUWE1 | CAGAGTTGGACAGAGTGAAA | TACACAGAGAGAGGAGGACA |

**Table S3: Parameters in model**

| Parameters | Definition | Value | Reference | |
| --- | --- | --- | --- | --- |
| $\bar{\boldsymbol{K}}$ | Effective bulk modulus of cytoplasm | 4.48 KPa | | [5] |
| $\bar{\boldsymbol{u}}$ | Effective shear modulus of cytoplasm | 1.29 KPa | | [5] |
| $\bar{\boldsymbol{\rho}_{\boldsymbol{0}}}$ | Effective contractility of cytoplasm | 3.15 KPa | | [5] |
| $\bar{\boldsymbol{K}_{\boldsymbol{\rho}}}$ | Effective modulus for motor density of cytoplasm | 3.65 KPa | | [5] |
| $\bar{\boldsymbol{\mu}_{\boldsymbol{\rho}}}$ | Effective modulus for polarization of cytoplasm | 0.90 KPa | | [5] |
| E_soft | Young’s modulus of soft matrix | 1.00 KPa | |  |
| E_stiff | Young’s modulus of stiff matrix | 20.00 KPa | |  |
| $\boldsymbol{\nu}$ | Poisson’s ratio of matrix | 0.30 | | [5] |
| E_nuclear | Young’s modulus of nuclei | 1.00 KPa | | [5] |
| $\bar{\boldsymbol{K}}\boldsymbol{\_cor}$ | Effective bulk modulus of cortex | 28.2 KPa | |  |
| $\bar{\boldsymbol{u}}\boldsymbol{\_cor}$ | Effective shear modulus of cortex | 8.10 Kpa | |  |
| $\bar{\boldsymbol{\rho}_{\boldsymbol{0}}}\boldsymbol{\_cor}$ | Effective contractility of cortex | 19.79 Kpa | |  |
| $\bar{\boldsymbol{K}_{\boldsymbol{\rho}}}\boldsymbol{\_cor}$ | Effective modulus for motor density of cortex | 27.37 Kpa | |  |
| $\bar{\boldsymbol{\mu}_{\boldsymbol{\rho}}}\boldsymbol{\_cor}$ | Effective modulus for polarization of cortex | 7.70 KPa | |  |

The differences in parameters of the cortex compared to the cytoplasm are all caused by the adjusted $\alpha_{v}=2.70 \mathrm{KPa}^{-1}$ and $\alpha_{d}=2.70 \mathrm{KPa}^{-1}$. $\alpha_{v}$: volumetric chemo-mechanical feedback parameter; $\alpha_{d}$: deviatoric chemo-mechanical feedback parameter[5].

**SI References**

[1] The Cancer Genome Atlas. Breast invasive carcinoma (BRCA) data, (2021). https://portal.gdc.cancer.gov/projects/TCGA-BRCA.

[2] B. Győrffy, Survival analysis across the entire transcriptome identifies biomarkers with the highest prognostic power in breast cancer, Computational and Structural Biotechnology Journal 19 (2021) 4101–4109. https://doi.org/10.1016/j.csbj.2021.07.014.

[3] Z. Tang, B. Kang, C. Li, T. Chen, Z. Zhang, GEPIA2: an enhanced web server for large-scale expression profiling and interactive analysis, Nucleic Acids Research 47 (2019) W556–W560. https://doi.org/10.1093/nar/gkz430.

[4] Uhlén, M., Fagerberg, L., Mjoberg, M., et al. (2015). Proteomics. The Human Protein Atlas. Retrieved from https://www.proteinatlas.org/, (n.d.).

[5] V.B. Shenoy, H. Wang, X. Wang, A chemo-mechanical free-energy-based approach to model durotaxis and extracellular stiffness-dependent contraction and polarization of cells, Interface Focus. 6 (2016) 20150067. https://doi.org/10.1098/rsfs.2015.0067.
